# Semantic interestingness measures for discovering association rules in the skeletal dysplasia domain

**DOI:** 10.1186/2041-1480-5-8

**Published:** 2014-02-05

**Authors:** Razan Paul, Tudor Groza, Jane Hunter, Andreas Zankl

**Affiliations:** 1 School of ITEE, The University of Queensland, St. Lucia, Queensland 4072, Australia; 2 Bone Dysplasia Research Group, UQ Centre for Clinical Research (UQCCR), The University of Queensland, Herston, Queensland 4006, Australia; 3 Genetic Health Queensland, Royal Brisbane and Women’s Hospital, Herston, Queensland 4006, Australia

## Abstract

**Background:**

Lately, ontologies have become a fundamental building block in the process of formalising and storing complex biomedical information. With the currently existing wealth of formalised knowledge, the ability to discover implicit relationships between different ontological concepts becomes particularly important. One of the most widely used methods to achieve this is association rule mining. However, while previous research exists on applying traditional association rule mining on ontologies, no approach has, to date, exploited the advantages brought by using the structure of these ontologies in computing rule interestingness measures.

**Results:**

We introduce a method that combines concept similarity metrics, formulated using the intrinsic structure of a given ontology, with traditional interestingness measures to compute semantic interestingness measures in the process of association rule mining. We apply the method in our domain of interest – bone dysplasias – using the core ontologies characterising it and an annotated dataset of patient clinical summaries, with the goal of discovering implicit relationships between clinical features and disorders. Experimental results show that, using the above mentioned dataset and a voting strategy classification evaluation, the best scoring traditional interestingness measure achieves an accuracy of 57.33%, while the best scoring semantic interestingness measure achieves an accuracy of 64.38%, both at the recall cut-off point 5.

**Conclusions:**

Semantic interestingness measures outperform the traditional ones, and hence show that they are able to exploit the semantic similarities inherently present between ontological concepts. Nevertheless, this is dependent on the domain, and implicitly, on the semantic similarity metric chosen to model it.

## Introduction

Over the course of the last decade, ontologies have become a fundamental building block in the knowledge acquisition and capturing processes in the biomedical domain. Repositories such as BioPortal [1] or the OBO Foundry [2] currently offer a varied range of ontologies, in addition to tool support to visualise, query and integrate concepts hosted by these ontologies. Subsequently, this enables the construction of decision support methods that use ontological background knowledge in order to produce more accurate and more refined outcomes.

Ontologies provide structured and controlled vocabularies and classifications for domain specific terminologies. Their adoption for annotation purposes provides a means for comparing medical concepts on aspects that would otherwise be incomparable. For example, the annotation of a set of disorders (directly or via patient cases) using a particular ontology enables us to compare these disorders, by looking at the underpinning annotation concepts. The actual comparison can be done in an exact or inexact manner. More concretely, one may take into account only those identical concepts that appear in all or some disorders, or may use a semantic similarity measure that relaxes the constraint on identical concepts. Such a semantic similarity measure represents a function that takes two or more ontology concepts and returns a numerical value that reflects the degree of similarity between these concepts in a given ontology. This comparison process represents a key aspect of typical data mining algorithms that form the core of any decision support method. For example, two ontological concepts, such as HP:0004481 (*Progressive macrocephaly*) and HP:0004482 (*Relative macrocephaly*) from the Human Phenotype Ontology (HPO) [3], would be treated differently by any classical data mining algorithm because of their symbolic (i.e., lexical grounding) difference. However, these two concepts, like any other two entities in an ontology, are to a certain extent semantically similar – a similarity that can be encoded via an existing or custom-made metric. Replacing exact matching with semantic similarity measures provides novel and exciting opportunities in knowledge discovery and decision support on annotated datasets [4-6].

Association rules [7] are valuable patterns that can discovered from annotated datasets. An association rule denotes an implication relationship (or a directed co-occurrence) between two sets of items within a transaction. A widely used algorithm to discover such association rules is Apriori [7]. However, regardless of the particular algorithm used, the discovery process has two major challenges: (i) too many rules may be generated (the rule *quantity* problem); (ii) not all rules are necessarily interesting (rule *quality* problem). The solution to the rule *quality* problem relies on specifying an interestingness measure [8-10] to encode the utility or significance of a pattern. These measures are intended for selecting and ranking patterns according to their potential interest and enables highly ranked rules to be immediately presented or used for particular purposes.

Existing work on interestingness measures takes into account only exact matching [10]. Semantic similarities, however, enable novel ways of interpreting data items, and hence may lead to the identification of association rules that are otherwise not discoverable via exact matching. In this manuscript, we advance the state of the art by exploring the application of semantic similarities in widely used interestingness measures in the context of association rule mining. In other terms, we aim to use existing taxonomic relations to calculate so-called “*semantic interestingness measures*”.

The context of our research is provided by the SKELETOME project [11], which aims to create a community-driven knowledge curation platform for the skeletal dysplasia domain. Skeletal dysplasias are a heterogeneous group of genetic disorders affecting skeletal development. Currently, there are over 450 recognised bone dysplasias, structured in 40 groups. Patients with skeletal dysplasias have complex medical issues including short stature, bowed legs, a larger than average head and neurological complications. Since most skeletal dysplasias are very rare (< 1:10,000 births), data on clinical presentation, natural history and best management practices is sparse. To date, we have developed an ontology, the Bone Dysplasia Ontology (BDO) [12], and a series of decision support methods [6,13]. BDO has been built using the latest nosology of bone dysplasias [14] that groups disorders according to their overlapping clinical and genetic features. For example, Achondroplasia and Diastrophic dysplasia are similar, and are both part of the FGFR3 Group, because they share a range of clinical features (i.e., short stature with very short arms and legs).

Within this manuscript, we investigate both traditional, as well as semantic interestingness measures in the context of association rule mining, to discover implicit relationships between clinical features and disorders in skeletal dysplasia domain. The main contributions of this work are the following: (i) firstly, we analyse which of the existing traditional interestingness measures enables a more accurate discovery of association rules in the skeletal dysplasia domain; (ii) secondly, we propose a series of interestingness measures based on semantic similarity metrics using existing ontologies as background knowledge; and (iii) finally, we perform an extensive empirical evaluation to measure the quality of the resulting rules, using an annotated dataset built on real patient data. At the same time, we show that, given an appropriate semantic similarity metric, the semantic interestingness measures outperform the traditional ones.

As already mentioned, our work focuses only on skeletal dysplasias, and hence it investigates the efficiency of the above-described methods only in this domain. However, the generic definition of a semantic interestingness measure proposed in this manuscript is directly applicable in any other domain, while the rest of the research can be used as a guideline for choosing an appropriate domain-specific semantic similarity metric to be applied as part of the overall measure.

## Background

This section provides an overview of the foundational blocks of the experiments performed in the context of our research. We start by introducing the Human Phenotype Ontology and the Bone Dysplasia Ontology – i.e., the ontologies used as background knowledge for the semantic similarity metrics. Then, we describe some of the basic notions of semantic similarities, and finally, we discuss some of the traditional interestingness measures.

### Human Phenotype Ontology

The Human Phenotype Ontology (HPO) [3] has lately become the de facto controlled vocabulary to capture and represent clinical and radiographic findings. The ontology consists of around 9,000 concepts describing modes of inheritance, onset and clinical disease courses and phenotypic abnormalities. This last category represents around 95% of the ontology and it is the main subject of our study. HPO structures phenotypic abnormalities in a hierarchical manner (via class-subclass relationships) from generic (e.g., HP:0000929 (*Abnormality of the skull*) to specific concepts (e.g., HP 0000256 – *Macrocephaly*). For instance, HP:0001629 (*Ventricular septal defect*) is a subclass of the concept HP:0010438 (*Abnormality of the ventricular septum*) in the sense that a ventricular septal defect is a kind of abnormality of the ventricular septum and hence. every person with a ventricular septal defect can also be said to have an abnormality of the ventricular septum. This goes along the line of the *True path rule*[15], which states that an annotation with a particular concept implies the path from that concept to the root to be “true”, or more concretely, a valid annotation with all ancestors of that concept.

One obvious advantage of capturing phenotypic information using ontologies is that it enables the design of association mining algorithms that can exploit the semantic relationships between concepts. For instance, an algorithm can be designed to support not only the patterns associated with a concept like HP:0001671 (*Abnormality of the cardiac septa*), but also those associated with its children, HP:0010438 (*Abnormality of the ventricular septum*) and HP:0011994 (*Abnormality of the atrial septum*).

### Bone Dysplasia Ontology

The International Skeletal Dysplasia Society (ISDS – http://www.isds.ch/) Nosology lists all recognised skeletal dysplasias and groups them by common clinical-radiographic characteristics and/or molecular disease mechanisms. The Nosology is revised every 4 years by an expert committee and the updated version is usually published in a medical journal. This is widely accepted as the “official” nomenclature for skeletal dysplasias within the biomedical community, with the latest version being published in 2010 [14].

The Bone Dysplasia Ontology [12] aims to complement the spectrum of existing ontologies and address the specific knowledge representation shortcomings of the ISDS Nosology. Its main role is to provide the scaffolding required for a comprehensive, accurate and formal representation of the genotypes and phenotypes involved in skeletal dysplasias, together with their specific and disease-oriented constraints. As opposed to the ISDS Nosology, the ontology enables a shared conceptual model, formalised in a machine-understandable description, in addition to a continuous evolution and a foundational building block for facilitating knowledge extraction and reasoning. Currently, the structure of the ontology follows closely the grouping of the disorders imposed by the expert committee via the Nosology by using class-subclass relationships between the 40 groups and their associated bone dysplasia members. These groups are then linked via the root concept Bone_Dysplasia.

### Semantic similarity

Annotations using Bio-ontologies allow us to compare concepts on various aspects by using their intrinsic semantic similarity. Semantic similarity represents the quantification of the degree of similarity between two or more ontological concepts. For example, the annotation of two bone dysplasias with concepts emerging from the same ontology, e.g., HPO, enables their comparison by looking at the semantic similarity between the concepts used for annotation. In addition to this implicit role, semantic similarity measures can also be used to discover association rules in annotated datasets.

In principle, there are two types of approaches for computing semantic similarity measures: node-based and edge-based. The former uses the nodes and their properties as the data source whereas the latter uses the edges between nodes and their associated types as data source. The node-based approaches usually rely on the notion of Information Content (IC) to quantify informativeness of a concept. An IC value of a node is calculated by computing the negative likelihood of its frequency in a large text corpora (*IC*(*c*)=-*log*(*p*(*c*))), with the intuition that the more probable is the appearance of a concept in a corpus, the less information it conveys.

A large number of node-based measures have been proposed using Information Content as a central element, some of the most widely used being listed below, i.e., Resnik [16], Lin [17] and Jiang and Conrath [18]. As a note, in the equations below, *MICA* denotes the Most Informative Common Ancestor, i.e., the common ancestor of the nodes with the highest Information Content.

(1) $$\begin{array}{c} \text{Resnik}:{\text{SIM}}_{\text{Res}}(c_{1},c_{2})=\text{IC}(c_{\text{MICA}}) \end{array}$$

(2) $$\begin{array}{c} \text{Lin}:{\text{SIM}}_{\text{Lin}}(c_{1},c_{2})=\frac{2*\text{IC}(c_{\text{MICA}})}{\text{IC}(c_{1})+\text{IC}(c_{2})} \end{array}$$

(3) $$\begin{array}{c} \text{Jiang and Conrath}:{\text{SIM}}_{\text{JC}}(c_{1},c_{2})\\ \;=1-\text{IC}(c_{1})+\text{IC}(c_{2})-\text{IC}(c_{\text{MICA}}) \end{array}$$

In the other category, i.e., edge-based approaches, Wu & Palmer [19] proposed a measure based on the length of the shortest path between the Least Common Ancestor (LCA) and path between each of the concepts and that common ancestor.

(4) $$\begin{array}{c} {\text{Dis}}_{W\&P}(c_{1},c_{2})=\frac{2*N3}{N1+N2+2*N3} \end{array}$$

where, *N*3 is the length of path from *LCA* to the root; *N*1 is the length of path from *c*_1_ to *LCA*; *N*2 is the length of path from *c*_2_ to *LCA*.

### Association rule mining

Association rules [7] provide knowledge in the form of probabilistic “if-then” statements, e.g., *I*→*Q*. The head of the association rule (i.e., the *if* part – *I*) is called antecedent, while the body (i.e., the *then* part – *Q*) is called consequent. The antecedent and consequent of an association rule are disjoint – they do not have any items in common. To express uncertainty in association rules, i.e., *I*→*Q* with a certain degree of certainty, several metrics can be used, two of the most widely adopted being *Support* and *Confidence* (discussed below). A set of association rules aimed for classification is called predictive association rule set. A class association rule set is a subset of association rules with the specified classes as their consequences. Predictive association rules form a small subset of class association rules. Generally, mining predictive association rules undergoes the following two steps: (i) Find all class association rules from a database, followed by (ii) Prune and organise the found class association rules to return a sequence of predictive association rules.

**Traditional interestingness measures.** As mentioned earlier, the rule discovery process is usually associated with two challenges, one of them being the rule *quality* problem, i.e., quantifying which of the discovered rules are more interesting. Interestingness measures play an important role in data mining, regardless of the kind of patterns being mined. They are intended for selecting and ranking patterns according to their potential interest to the user. Below, we present a number of existing association rules interestingness measures [10], which we have also applied in our experiments. This set of measures rely on the foundational *Support* and *Confidence* metrics.

Let *T*={*t*_1_,*t*_2_,…,*t*_*n*_} be a database of *n* transactions with a set of attributes (or items) *I*={*i*_1_,*i*_2_,…,*i*_*m*_}. For an itemset *I*_*X*_⊆*I* and a transaction *t*∈*T*, we say that *t* supports *I*_*X*_ if *t* has values for all the attributes in *I*_*X*_. By $T_{I_{X}}$ we denote the transactions that contain all attributes in *I*_*X*_.

The *Support* of *I*_*X*_ is computed as

(5) $$\begin{array}{c} \text{Support}(I_{X})=\frac{T_{I_{X}}}{n} \end{array}$$

or the fraction of transactions that include all attributes in *I*_*X*_.

The *Confidence* of an association rule *I*_*X*_→*Q*, where Q is also an itemset (*Q*⊂*I*) and *Q*∩*I*_*X*_=*ϕ*, is defined by:

(6) $$\text{Confidence}(I_{X}\to Q)=\frac{\text{Support}(I_{X},Q)}{\text{Support}(I_{X})}$$

or the ratio between the number of transactions that include all items in the consequent (*Q*), as well as in the antecedent (*I*_*X*_) – namely, the *Support* of the union of *I*_*X*_ and *Q* – and the number of transactions that include all items in the antecedent (i.e., the *Support* of *I*_*X*_).

Confidence alone may not be enough to assess the descriptive interest of a rule, as rules with high confidence may occur by chance. Such spurious rules can be detected by determining whether the antecedent and the consequent are statistically independent. This inspired a number of measures, including Lift, Conviction, Leverage, Jaccard, Cosine and Correlation Coefficient [8-10]. We provide their mathematical definitions in the following sections.

## Materials and methods

### Annotation dataset

The rare nature of bone dysplasias makes the data collection particularly challenging. In 2002, the European Skeletal Dysplasia Network (ESDN, http://www.esdn.org/) was created to alleviate, at least partly, the data sparseness issue. At the same time it aimed to provide a collaborative environment to help with the diagnosis of skeletal dysplasias and to improve the information exchange between researchers. To date, ESDN has gathered over 1,200 patient cases, which have been discussed by its panel of experts. The ESDN case workflow consists of three major steps: (i) a patient case is uploaded and an initial diagnosis is set by the original clinician that referred the case; (ii) the panel of experts discusses the case until an agreement is reached; (iii) the panel of experts recommends a final diagnosis. Among the total number of cases, 744 have a final bone dysplasia diagnosis (the remaining cases were not thought to be true bone dysplasias by the experts), with a total of 114 different skeletal dysplasias covered.

Patient clinical summaries in ESDN are represented in a free text format. The language used within the ESDN clinical summaries suffers from several issues, such as synonymy (several terms having the same meaning) or hyponymy (one term being more specific than another). In order to be able to use this data, we extracted patient phenotypes by annotating the text with corresponding terms from the Human Phenotype Ontology (HPO). The actual annotation process was performed using the National Centre for Biomedical Ontology (NCBO) Annotator [20], an ontology-based web service for annotation of textual sources with biomedical concepts. A bone dysplasia expert (one of the co-authors) has manually validated the resulting HPO annotations to ensure their correctness and to eliminate, in particular, false positives. As a remark, the false negatives resulted from the annotation process may be under-estimated, and could not be validated since we were not able to perform a full-fledged annotation of the clinical summaries. The diagnosis associated with the patient cases has also been annotated with concepts from the Bone Dysplasia Ontology (BDO). More concretely, the final diagnosis set by the panel of experts has been converted to the corresponding BDO concept.

In order to achieve realistic results using association rule mining, from the 114 types of dysplasias present in the ESDN dataset, we chose only those that were represented by more than 10 patient cases. This has reduced our dataset to 394 annotated patient cases (i.e., around 33% of the total number) diagnosed with 15 different bone dysplasias. The set features a total of 441 distinct phenotypes, with an average of 63.67 distinct phenotypes per disorder and an average of 4.49 distinct phenotypes per case. The experiments described in this manuscript use this dataset for training and testing purposes.

### Proposed approach

Our goal is to discover association rules from annotated and diagnosed patient cases in order to observe co-occurrence relationships between clinical features and disorders. In other words, we aim to find association rules of the form {*I*_*CF*_}→{*I*_*BD*_}, where *I*_*CF*_ represents the set of clinical features of a patient and *I*_*BD*_ is a bone dysplasia diagnosis. From a conceptual perspective, *I*_*CF*_ will comprise annotations assigned to patient cases, or more concretely, HPO concepts. We have adapted the Apriori algorithm by adding two constraints, required to match our aim: (i) every desired itemset must have one set of clinical features and a single dysplasia, and (ii) both candidate itemsets and frequent itemsets can have at most one dysplasia item.

Following the discovery of the desired itemsets, these are partitioned into two components: a component containing the skeletal dysplasia and one containing the phenotypes. A Boolean function that determines the type of a component is used to perform this classification. Subsequently, we calculate the different traditional or semantic interestingness measures between the bone dysplasia component and the phenotype set of the rule.

### Modelling traditional support in the context of semantic annotations

If an itemset consists of the items *I*={*i*_1_,*i*_2_,*i*_3_,…,*i*_*m*_} for the reference concept *RC* and there are *n* transactions in the knowledge base *KB*, *Support* is defined as the proportion of instances of the reference concept *RC* in the knowledge base which contain the itemset *I*.

(7) $$\begin{array}{c} \text{Support}(I,\text{RC},\text{KB})\\ \;=\frac{\text{Number of instances of concept RC that contain the itemset I}}{\text{The total number of instances of the concept RC}} \end{array}$$

In our case, the reference concept (*RC*) is represented by the patient (*P*) and *KB* is annotated dataset. Below we present an example of traditional *Support* calculation.

Let us consider the following set of clinical features represented by HPO concepts (*cf*∈*I*_*CF*_), in addition to a bone dysplasia: 

• *cf*_1_ – HP:0008921 (*Neonatal short-limb short stature*)

• *cf*_2_ – HP:0008905 (*Rhizomelic short stature*)

• *cf*_3_ – HP:0000772 (*Abnormality of the ribs*)

• *cf*_4_ – HP:0000774 (*Narrow chest*)

• *bd*_1_ – BDO:Achondroplasia

Let us also consider three reference concepts (i.e., patients) *p*_1_, *p*_2_ and *p*_3_ and assume that the *KB* contains the following itemsets: 

• $I(p_{1})=\{I_{{\text{cf}}_{1}}(p_{1}),I_{{\text{cf}}_{3}}(p_{1}),{\text{bd}}_{1}\}$

• $I(p_{2})=\{I_{{\text{cf}}_{1}}(p_{2}),I_{{\text{cf}}_{4}}(p_{2}),{\text{bd}}_{1}\}$

• $I(p_{3})=\{I_{{\text{cf}}_{2}}(p_{3}),I_{{\text{cf}}_{3}}(p_{3}),{\text{bd}}_{1}\}$

where $I_{{\text{cf}}_{x}}(p_{x})\;=\;\{{\text{cf}}_{x}|\text{exhibits}(p_{x},{\text{cf}}_{x})\}$. Our goal is to compute the support of the itemset $I(p)\;=\;\{I_{{\text{cf}}_{1}}(p),I_{{\text{cf}}_{3}}(p),{\text{bd}}_{1}\}$. We can quickly observe that there is one patient instance that contains this pattern – i.e., *p*_1_. Since the total number of patient instances is 3, traditional support is then:

(8) $$\text{Support}(I,P,\text{KB})=\frac{1}{3}=0.33$$

However, a close look at *cf*_1_ and *cf*_2_ in HPO reveals that these concepts are fairly similar (they have a direct common ancestor in HP:0008873 – *Disproportionate short-limb short stature*), but not exactly the same. *cf*_3_ and *cf*_4_ are in a similar situation, with the parent of HP:0000774 (i.e., HP:0005257 – *Thoracic hypoplasia*) being a sibling of *cf*_3_. Unfortunately, traditional *Support* cannot leverage this semantic similarity information as it relies on exact matching. To overcome this issue, we propose an alternative set of semantic interestingness measures (*Semantic Support*, *Semantic Confidence*, etc.).

### Semantic similarity of items

Our intuition is that by using semantic similarity measures on patient findings (i.e., HPO concepts) we are able to leverage and use the semantic relationships between phenotypes that cannot, otherwise, be acquired by typical data mining processes (due to their term-based matching process). As an example, if the background knowledge base lists HP:0000256 (*Macrocephaly*) as a phenotype of *Achondroplasia* and a new patient exhibits HP:0004439 (*Craniofacial dysostosis*), we want to use the semantic similarity value between the two concepts to associate the later to *Achondroplasia* with a certain probability. The semantic similarity between the concepts could be inferred, for example, via their most common ancestor – HP:0000929 (*Abnormality of the skull*). Such an association is not possible when employing a typical data mining process since each term would be considered individually and only in the context provided by the background knowledge base.

In principle, a good semantic similarity measure needs to take into account the specific aspects of the target domain. There are, nevertheless, a series of requirements – emerging also from the bone dysplasia domain and the structure of HPO – that are generally applicable: 

• Given two HPO concepts, we consider them to be more similar if they are closer to each other (i.e., the path between them is shorter). E.g., HP:0004481 (*Macrocephaly progressive*) will be considered more similar to HP:0000256 (*Macrocephaly*) than HP:0004488 (*Macrocephaly at Birth*), because the distance between HP:0004481 and HP:0000256 is 1 whereas the distance between HP:0004481 and HP:0004488 is 2.

• Several strategies have been used in choosing the semantic similarity function. Li et al. [21], in their work on modelling and capturing semantic similarity in WordNet, have employed an exponent function to transfer the path length between concepts into a similarity value and have showed that the exponential measure significantly outperforms traditional similarity measures. Given that the design philosophy of HPO and WordNet are similar, we derive the similarity between two phenotypes as an exponent function of the path length between their corresponding HPO concepts. The same rationale is valid also for BDO.

• In order to be able to calculate the semantic interestingness measures, semantic similarity needs to take values between 0 to 1. At the same time, an exact match should be signalled by a semantic similarity value of 1.

• The semantic similarity value of two concepts should be dependent on the specificity of their LCA (i.e., its location in the overall hierarchy). More concretely, we consider the more specific LCA to be more informative. E.g., HP:0004439 (*Craniofacial dysostosis*) (as an LCA) should be considered more informative than HP:0000929 (*Abnormality of the skull*), which is in this case, is its direct parent.

In the following we describe a set of domain-oriented semantic similarity functions that satisfy the above-listed requirements.

**Domain-specific semantic similarity measures.** If *i*_1_ and *i*_2_ are two items, we define the semantic similarity between them as:

(9) $$\begin{array}{c} \text{SemSim}(i_{1},i_{2})=\frac{\text{Dist}(\text{LCA}(i_{1},i_{2}),\text{Root})}{\text{Dist}(i_{1},i_{2})+\text{Dist}(\text{LCA}(i_{1},i_{2}),\text{Root})} \end{array}$$

where *Dist*(*LCA*(*i*_1_,*i*_2_),*Root*) is the length of path from *LCA*(*i*_1_,*i*_2_) to the root and *Dist*(*i*_1_,*i*_2_) is a distance measure between *i*_1_ and *i*_2_ that depends on the underlying types of the items.

If the items under scrutiny are phenotypes, we define *Dist*(*i*_1_,*i*_2_) as shown in Eq. 10.

(10) $$\text{Dist}(i_{1},i_{2})=\left\{\begin{array}{cc} 2^{l_{x}}, & \;\text{if}i_{1}\neq i_{2}\\ 0, & \;\;\text{if}i_{1}=i_{2}\neq \text{root}\\ 1, & \;\;\text{if}i_{1}=i_{2}=\text{root} \end{array}\right)$$

where *l*_*x*_ is the shortest path between *i*_1_ and *i*_2_. This formula determines the semantic similarity of two HPO terms based on both the distance between these terms and the location of their LCA in the HPO structure. It can also be observed that the larger the distance between the terms, the less similar they will be. Finally, if two concepts are the same but do not denote the root, the value of the function is 0, while if they do denote the root, the value of the function is 1, to avoid the division by 0 case.

In Eq. 10 the shortest path length is scaled by an exponential function to provide more weight to distance rather than depth. Furthermore, the base and the exponent of this power function aim to overemphasise the similarity between phenotypes when taking into account the HPO structure. Generally, this similarity decreases faster than the distance. For instance, the distance between *Macrocephaly* and *Macrocephaly progressive* is 1 and they are very similar, while the distance between *Abnormality of Skull* and *Macrocephaly progressive* is 3, with the former being much more generic and different to *Macrocephaly progressive* than any of the other macrocephalies.

Similar to the phenotype distance described above, if we consider two disorders using the Bone Dysplasia Ontology, we define the same *Dist*(*i*_1_,*i*_2_) as shown in Eq. 11 – the semantic similarity equation remains unchanged (i.e., as per Eq. 9).

(11) $$\text{Dist}(i_{1},i_{2})=\left\{\begin{array}{cc} 10^{l_{x}-2}, & \;\text{if}i_{1}\neq i_{2}\\ 0, & \;\;\text{if}i_{1}=i_{2}\neq \text{root}\\ 1, & \;\;\text{if}i_{1}=i_{2}=\text{root} \end{array}\right)$$

where *l*_*x*_ is again the shortest path between *i*_1_ and *i*_2_.

The rationale behind Eq. 11 is the same as for Eq. 10 (see above), with the remark that the overall similarity between disorders decays at an even higher rate (with the distance in BDO) because of their coarse grained nature, which has led to a fairly flat structure of the ontology. The structure of the ontology, and more concretely its maximum depth (i.e., 2), has influenced the constant (*2*) in the exponent of the formula (*l*_*x*_-2). The intuition is that concepts that belong to the same group, i.e., they are at the second level in the hierarchy and the distance between them is 2 (via the LCA), should receive the highest similarity, after the exact match.

### Semantic support

Given a knowledge base and an itemset, our goal is to automatically derive a score that indicates the proportion of transactions in the knowledge base that contain the itemset at a semantic level, thus going beyond the exact matching methods traditionally used for this task. This needs to take into account the relations between items. We attempt to model the semantic support of an itemset as a function of the semantic similarity of the terms present in the knowledge base and the itemset.

If we consider a database *T* with *n* transactions {*t*_1_,*t*_2_,…,*t*_*n*_} and *m* items {*i*_1_,*i*_2_,…,*i*_*m*_}, *Semantic Support* of {*i*_1_,*i*_2_,…,*i*_*p*_} (*p*≤*m*) is calculated as follows:

(12) $$\begin{array}{c} \;\text{SemSupport}(i_{1},i_{2},\ldots ,i_{p})=\frac{1}{n}\;*\;\overset{n}{\underset{q=1}{\sum }}\overset{p}{\underset{j=1}{\prod }}\underset{v=1\text{to}|t_{q}|}{arg max}\;||\text{SemSim}(\;i_{j},i_{v}\;)|| \end{array}$$

The value of the Semantic Similarity (*SemSim*) ranges from 0 to 1 and so does the value of the *Semantic Support*.

### Semantic interestingness measures

Semantic interestingness measures take into account how data items are semantically related. To do so, it makes use of the underlying structure of the ontology that hosts the corresponding items (e.g. generalisation, specialisation, etc). Hence, if we replace the traditional *Support* element in the confidence calculation with *Semantic Support* we get *Semantic Confidence*. The same process can be applied for the other well-known interestingness measures, such as lift, conviction, etc. Below we list the corresponding semantic calculation for these measures for an association rule *I*_*X*_→*Q*.

(13) $$\begin{array}{c} \;\text{SemConfidence}(I_{X}\to Q)=\frac{\text{SemSupport}(I_{X},Q)}{\text{SemSupport}(I_{X})} \end{array}$$

(14) $$\begin{array}{c} \;\text{SemLift}(I_{X}\to Q)=\frac{\text{SemConfidence}(I_{X},Q)}{\text{SemSupport}(Q)} \end{array}$$

(15) $$\begin{array}{c} \;\text{SemConviction}(I_{X}\to Q)=\frac{1-\text{SemSupport}(Q)}{1-\text{SemConfidence}(I_{X}\to Q)} \end{array}$$

(16) $$\begin{array}{c} \text{SemLeverage}(I_{X}\to Q)=\text{SemSupport}(I_{X},Q)\\ \;-\text{SemSupport}(I_{X})*\text{SemSupport}(Q) \end{array}$$

(17) $$\begin{array}{c} \text{SemJaccard}(I_{X}\to Q)\\ \;=\frac{\text{SemSupport}(I_{X},Q)}{\text{SemSupport}(I_{X})+\text{SemSupport}(Q)-\text{SemSupport}(I_{X},Q)} \end{array}$$

(18) $$\begin{array}{c} \;\text{SemCosine}(I_{X}\to Q)=\frac{\text{SemSupport}(I_{X},Q)}{\sqrt{\text{SemSupport}(I_{X})*\text{SemSupport}(Q)})} \end{array}$$

(19) $$\begin{array}{c} \text{SemCorrelationCoeff}(I_{X}\to Q)\\ =\frac{\text{SemLeverage}(I_{X}\to Q)}{\sqrt{S\;\text{Supp}(I_{X})\;*\;S\;\text{Supp}(\;Q)\;*\;(\;1-S\;\text{Supp}(I_{X}\;)*(\;1-S\;\text{Supp}(Q)\;)}} \end{array}$$

*SSupp* in Eq. 19 denotes *Semantic Support*.

### Experimental design

We have carried out a series of experiments with the following goals: 

• Firstly, we aim to analyse the accuracy of the resulting association rules when using existing traditional interestingness measures;

• Secondly, we are interested in finding out the same accuracy, but when using the proposed semantic interestingness measures;

• Finally, we aim to observe the difference between the accuracies produced via the two methods.

The quality of discovered rules depends on their ability to determine the correct diagnosis. To measure accuracy, we have employed a voting strategy, which is described below.

The purpose of evaluating the discovered rules is to understand the utility of the interestingness measures. Voting allows all firing association rules to contribute to the final prediction. This strategy combines the associations *KF*(*p*_*x*_) that fire upon a new patient case *p*_*x*_. A simple voting strategy considers all the rules in *KF*(*p*_*x*_), groups the rules by antecedent, and for each antecedent *I*_*X*_ obtains the class corresponding to the rule with highest confidence. We will denote the class voted by an antecedent *I*_*i*_ with a binary function *vote*(*I*_*i*_,*bd*) that takes the value 1 when *I*_*i*_ votes for disorder *bd*, and 0 for the any other class – {*bd*_*n*_1,*bd*_2_,…,*bd*_*n*_}∈*BD* represent a set of bone dysplasias. The disorder that receives the maximum vote is the most probable diagnosis for patient case *x*.

(20) $$\text{TotalVote}({\text{bd}}_{i})=\underset{I_{i}\in \text{antecedents}(\text{KF}(p_{x}))}{\sum }\text{Vote}(I_{i},{\text{bd}}_{i})$$

Weighted voting is similar to simple voting, however, each vote is multiplied by a factor that quantifies the quality of the vote. In the case of association rules, this can be done using one of the above defined measures.

(21) $$\begin{array}{c} \;\text{TotalVote}({\text{bd}}_{i})=\;\;\;\underset{I_{i}\in \text{antecedents}(\text{KF}(p_{x}))}{\sum }\;\;\;\text{Vote}(I_{i},{\text{bd}}_{i})*\text{QVote}(I_{i},{\text{bd}}_{i}) \end{array}$$

In our case, *QVote*(*I*_*i*_,*bd*_*i*_) is the quality of vote, or more concretely the maximum interestingness of that particular antecedent group.

We have performed individual experiments for each of the interestingness measures previously described, using the voting strategy. To assess their efficiency, we have calculated the overall accuracy of the discovered association rules. In all experiments, we compute the prediction accuracy as the overall percentage of correctly predicted disorders at a given recall cut-off point (i.e., by taking into account only the top K predictions for different values of K, where K is the recall cut-off point). Hence, a success represents a correctly predicted disorder (the exact same, and not a sub or super class of it), while a miss represents an incorrectly predicted disorder. If *N* is the total number of test cases and *C*_*P*_ is the number of correctly predicted disorders, then *Accuracy*=*C*_*P*_/*N*. This is expressed in percentages in Tables 1, 2 and 3 in the Results section.

**Table 1:** Experimental results on finding the quality of association rules, discovered using traditional interestingness measures

**Traditional**	**Accuracy**	**Accuracy**	**Accuracy**	**Accuracy**	**Accuracy**
**interestingness measures**	**K = 1**	**K = 2**	**K = 3**	**K = 4**	**K = 5**
Confidence	28.77	**46.58**	**53.42**	**54.79**	**57.33**
Lift	26.03	36.99	42.47	49.32	57.53
Conviction	28.77	43.84	46.58	49.32	57.53
Correlation coefficient	27.40	36.99	45.21	52.05	57.53
Cosine	28.76	43.84	49.31	54.79	58.90
Jaccard	**31.51**	45.21	52.05	**54.79**	**57.53**
Leverage	24.66	35.62	46.58	54.79	57.53

The voting strategy has been used as classification method and the association rules have been used as background knowledge.

**Table 2:** Experimental results on finding the quality of association rules, discovered using semantic interestingness measures

**Semantic**	**Accuracy**	**Accuracy**	**Accuracy**	**Accuracy**	**Accuracy**
**Interestingness measures**	**K = 1**	**K = 2**	**K = 3**	**K = 4**	**K = 5**
Semantic confidence	31.51	**49.32**	**57.53**	**61.64**	**64.38**
Semantic lift	27.40	38.36	47.95	57.53	61.64
Semantic conviction	32.88	43.84	53.42	56.16	58.90
Semantic correlation coefficient	23.29	38.36	45.21	57.53	64.38
Semantic cosine	31.51	47.95	52.05	57.53	61.64
Semantic jaccard	**34.25**	46.58	56.16	**61.64**	**64.38**
Semantic leverage	26.02	36.99	53.42	58.90	63.01

The voting strategy has been used as classification method and the association rules have been used as background knowledge.

**Table 3:** Comparative overview of the experimental results achieved by the traditional and semantic interestingness measures

**Interestingness**	**Accuracy**	**Accuracy**	**Accuracy**	**Accuracy**	**Accuracy**
**measures**	**K = 1**	**K = 2**	**K = 3**	**K = 4**	**K = 5**
Traditional	28.77	46.58	53.42	54.79	57.53
Semantic	31.51	49.32	57.53	61.64	64.38

As mentioned earlier in the manuscript our annotated dataset consisted of over 300 patience cases, with the clinical features annotated using HPO and the disorders using BDO. In order to provide an accurate view over the prediction of the discovered rules, each experiment has been performed as a 5-fold cross validation with an 80-20 split (80% knowledge base, 20% test data). Tables 1, 2 and 3 lists the resulted average accuracy at five different recall cut-off points.

Within each experiment, we have used a relatively low minimum *Support* of 5/*N*, where *N* is the total number of cases, because we are interested in extracting both frequent and occasional associations. Every rule was able to contribute to the voting. Controlling the number of rules using any minimum interestingness threshold can bias the voting and hence, the overall result. Consequently, we have not used this parameter to control the number of rules. Finally, we have used a maximum itemset size of 10 as the computational cost increases exponentially with the itemset size in the association rule mining process.

## Results

In this section we present and discuss the experimental results achieved using traditional and semantic interestingness measures. We start with the semantic similarity proposed in the previous sections and then compare its results against a series of classic semantic similarity measures.

### Proposed semantic similarity metric

In order to observe the quality improvements brought by semantic interestingness measures over the traditional ones, we have evaluated the discovered rules against real world patient data. As already mentioned, we performed two sets of experiments. Firstly, we have compared and evaluated different traditional interestingness measures. Then, we performed the same experiment but by using semantic interestingness measures. This has enabled us to perform an overall comparison between the two types of measures.

Table 1 lists the experimental results for the traditional measures. A first observation is that Confidence has the overall best behaviour. At any recall cut-off point greater than 2 (K > 1) Confidence outperforms or scores similarly to the other measures. For example, it achieves an accuracy of 46.58% for K = 2 and 53.42% for K = 3, both with 1.37% higher than the second scoring measure, Jaccard. The only exception appears for K = 1, where Jaccard outperforms Confidence by 2.74%. A second, interesting, observation is that with the increase in the recall cut-off point, the measures reach a common ground, and hence, achieve the same performance – for K = 5, six of the seven measures score the same accuracy (57.53%).

Each of the measures we have considered in our experiments studies certain properties of the data. Consequently, the above-listed results enable us to reach a better understanding of the underlying nature of the relationships manifested by the data in our bone dysplasia annotated dataset. For example, Confidence measures the level of causality (implication), while Jaccard measures the degree of overlap among the given sets, or in our cases patient phenotypes. This leads to the conclusion that the bone dysplasia data seems to be governed more by causality and overlap, rather than, for example, co-occurrence, which is described by Lift.

Table 2 lists the experimental results for the semantic interestingness measures. We can easily observe that the results follow the same trend as in the previous experiment. Semantic Confidence has, again, an overall best behaviour for K > 1, outperforming Semantic Jaccard with 1.37% for K = 2 (49.32%) and K = 3 (57.53%) and achieving the same accuracy for K = 4 (61.64%) and K = 5 (64.38%). Semantic Jaccard achieves a better accuracy for K = 1, i.e., 34.25%, with 2.74% higher than Semantic Confidence. Finally, as in the previous experiment, we observe that the increase in the recall cut-off point leads to a more uniform accuracy across all measures, although slightly less aligned as they do not achieve the exact same accuracy.

A comparative overview of the two types of measures is presented in Table 3, where we can observe that semantic measures achieve better results than the traditional ones. Furthermore, the increase in the recall cut-off point leads to a bigger difference in accuracy, from 2.74% for K = 1 to 6.85% for K = 5.

The main reason behind the increase in accuracy is the use of similarity matching between terms. For instance, an ESDN patient diagnosed with Achondroplasia had the following phenotypes: *Rhizomelic short stature*, *Muscular hypotonia*, *Hypoplasia involving bones of the extremities* and *Malar flattening*. The classifier using traditional confidence measures was not able to classify correctly this case, while the classifier using semantic confidence did. The semantic similarity employed by the latter found an association between *Rhizomelic short stature* and Achondroplasia based on the more generic *Short stature* phenotype, which is common in Achondroplasia. This represents a clear example where the exact matching used by traditional classifiers fails. Another similar instance was in the case of a MED patient that exhibited the following phenotypes: *Pes planus* (i.e., flat feet), *Rhizomelic shortening* and *Frontal bossing*. As in the previous example, the classifier using traditional confidence failed to classify this instance correctly, while the one using semantic confidence did, based on the semantic similarity between *Pes planus* and the diverse feet abnormalities that characterise MED.

In order to have an accurate view over the classification results, we have checked the statistical significance of the increase in accuracy at recall cut-off point 5. The purpose of this statistical significance testing was to assess the performance of the classification using semantic rules against the performance of the classification using traditional rules, both on the ESDN dataset. Such a test would validate the observed increase in accuracy of 6.85% and would show that it has not been obtained by chance.

Since the comparison is between two different approaches on a single domain (skeletal dysplasias), we have used the McNemar’s Chi-squared test with continuity correction [22]. The null hypothesis was that the number of patient cases correctly classified by the classifier using semantic confidence but not by the one using traditional confidence is equal to the number of patient cases correctly classified by the classifier using traditional confidence but not by the one using semantic confidence. Table 4 shows the distribution of the 394 patient cases used in our experimental classification setting: (i) 205 patient cases were correctly classified by both classifiers; (ii) 118 patient cases were misclassified by both classifiers; (iii) 51 patient cases were correctly classified using semantic confidence; and (iv) 20 patient cases were correctly classified using traditional confidence. From this data, the McNemar test statistic with continuity correction is:

**Table 4:** Distribution of classification results in the McNemar’s statistical significance test

		**Semantic confidence based**		
		**classifier**		
		**Positive**	**Negative**	**Total**
**Traditional confidence based classifier**	Positive	205	20	225
	Negative	51	118	169
	Total	256	138

(22) $$\begin{array}{c} \chi_{\text{McNemar}}^{2}=\frac{{\left(|51-20|-1\right)}^{2}}{51+20}=12.67 \end{array}$$

A McNemar test value of 12.67 corresponds to a p-value of 0.00037157, which provides strong evidence to reject the null hypothesis. We can, hence, conclude that the semantic interestingness measures we have proposed are able, with the help of the underlying domain ontologies, to take advantage of the similarity matching between the terms in the skeletal dysplasia domain.

### Classic semantic similarity metrics

In order to understand the role carried by the semantic similarity metric in the classification based on semantic interestingness we have experimented with three classic semantic similarities, defined earlier in the paper: Resnik, Lin and Wu & Palmer. The results achieved by each of these metrics are discussed below.

Table 5 lists the experimental results achieved by the semantic interestingness measures employing Resnik as semantic similarity. A first observation is that all measures have performed uniformly, while from a comparative perspective, they performed worse than exact matching and our proposed semantic similarity method. As in the previous experiments, we observe that the increase in the recall cut-off point leads to a more uniform accuracy across all measures. The Resnik semantic similarity method is primarily dependent on the frequency of the most informative common ancestors. If any of the ancestors does not exist in the corpus, the similarity value becomes infinity, i.e., the concepts under scrutiny are completely dissimilar. In the case of our dataset, this is the main issue behind the failure of the Resnik semantic similarity – being a real-world dataset, most patient cases will feature concrete (very specific) phenotypes, while common ancestors represent more generic/abstract concepts rarely found in clinical summaries. For example, the semantic similarity of *Dolichocephaly* and *Full cheeks* is *∞*, due to the fact that the frequency of all their ancestors (*Abnormality of the head*, *Abnormality of head and neck* and *Phenotype abnormality*) in the patient cases is 0.

**Table 5:** Experimental results on finding the quality of association rules discovered using semantic Interestingness measures that employed Resnik as semantic similarity method

**Semantic interestingness measures**	**Accuracy**	**Accuracy**	**Accuracy**	**Accuracy**	**Accuracy**
**(Employing Resnik)**	**K = 1**	**K = 2**	**K = 3**	**K = 4**	**K = 5**
Semantic confidence	5.48	6.85	9.59	10.96	10.96
Semantic lift	5.48	8.22	9.59	9.59	10.96
Semantic conviction	2.74	6.85	9.59	9.59	10.96
Semantic correlation coefficient	5.48	8.22	9.59	9.59	10.96
Semantic cosine	5.48	8.22	9.59	10.96	10.96
Semantic jaccard	5.48	8.22	9.59	9.59	10.96
Semantic leverage	5.48	8.22	9.59	9.59	10.96

The experimental results for the semantic interestingness measures using the second semantic similarity – Lin – have led 0% accuracy on all measures and all five recall cut-off points – consequently we have have included them in a table. As in the case of Resnik, Lin is also heavily dependent on the IC of the common ancestors, and hence suffers from the same issue discussed above. Another problematic aspect of the Lin measure is that, in the context of the ESDN data, it assigns higher similarity values to partial matches than to exact matches. A similarity value of 1 is achieved when the concepts being measured are the exact same – e.g., *Short long bones*. However, when the concepts are different and any of their ancestors is present in the underlying corpus, the similarity value will, usually, be greater than 1. This is because the frequency of the ancestors (more abstract concepts) will be less than the frequency of the actual concepts and IC is inversely proportional to frequency.

For instance, the semantic similarity value between *Macrocephaly* and *Hypoplasia involving bones of the extremities* is 2.19 because the frequency of their most informative common ancestor – *Abnormality of the skeletal system* is less than that of both concepts. The latter occurs only 5 times in the corpus whereas *Macrocephaly* and *Hypoplasia involving bones of the extremities* occur 41 and 70 times, respectively. The Resnik measure is able to avoid this issue by treating exact and partial matches in the same manner – i.e., directly and only via the IC of the most informative common ancestor and not by further diving it by the IC of the actual concepts. In an ideal scenario, exact matches should assign higher similarity values that partial matches.

Finally, Table 6 lists the experimental results for the semantic interestingness measures using the last semantic similarity – Wu & Palmer. We can observe that the results follow fairly closely the trend present in our experiments with the traditional interestingness measures and the semantic interestingness measures employing our proposed metric. Similarly to those results, there is an increase in accuracy with the increase in the recall cut-off point, which also leads to a more uniform accuracy across all measures. Semantic Confidence has an overall best behaviour for K > 1, while Semantic Leverage achieves a better accuracy for K = 1, i.e., 23.29%, with 2.74% higher than Semantic Confidence.

**Table 6:** Experimental results on finding the quality of association rules discovered using semantic Interestingness measures that employed Wu & Palmer as semantic similarity method

**Semantic interestingness measures**	**Accuracy**	**Accuracy**	**Accuracy**	**Accuracy**	**Accuracy**
**(Employing Wu and Palmer)**	**K = 1**	**K = 2**	**K = 3**	**K = 4**	**K = 5**
Semantic confidence	20.55	**35.62**	**36.99**	**42.47**	**54.79**
Semantic lift	13.70	26.03	28.77	39.73	52.05
Semantic conviction	16.44	24.66	26.03	34.25	52.05
Semantic correlation coefficient	20.55	28.77	32.88	39.73	43.84
Semantic cosine	21.92	32.88	34.25	42.47	54.79
Semantic jaccard	20.55	35.62	38.36	41.10	54.79
Semantic leverage	**23.29**	30.14	32.88	38.36	45.21

The Wu & Palmer similarity score ranges between 0 and 1, with 1 denoting an exact match and the rest of the values being assigned based on the depth in the hierarchy and distance between the concepts. This is the main reason behind its good performance – i.e., it uses only structural distances instead of information content. It is, however, biased more towards depth than the actual distance between concepts, or more concretely it is influenced by the depth of the common ancestor of the concepts. In the case of out dataset, and using HPO as background knowledge, this represents an issue because most common ancestors are located at fairly uniform depths (due to the inherent specificity of the terms) and, as such, do not provide enough variety for the final similarity score.

In conclusion, none of the classic semantic similarities perform better than the approach we have proposed: node-based similarities are heavily influenced by the presence, or more precisely absence, of the common ancestor in the dataset (which leads to complete dissimilarity), while the edge-based similarity we have experimented with focuses more on the depth of the common ancestor, as opposed to the distance between the concepts, which is more appropriate given our dataset and background knowledge.

## Discussion and conclusions

### Main findings

In conclusion, based on the annotated bone dysplasia dataset, Confidence appears to be the best interestingness measure regardless of way in which is computed, i.e., traditional or semantic. The use of semantics provides a marginal, but consistent, improvement in accuracy over traditional measures. Since the semantic similarity relies on the structure of the underlying ontology, this improvement is heavily dependent on the reflection provided by the domain ontology over the real domain knowledge.

### Limitations and generalisation

Every domain is governed by a set of rules. A good semantic similarity measure needs to take into account the rules of the target domain. In our case, we have proposed and used two particular similarity measures, one tailored on the knowledge externalised by HPO and one on the structure of bone dysplasias, provided by BDO. These semantic similarity measures are not necessarily directly applicable to other domains. Consequently, while the definition of *semantic support* is generic, in order to apply our approach in a different domain, an investigation is required to determine the most appropriate semantic similarity for that domain.

### Related work

The literature contains a number of studies on using association rule mining to identify relationships among medical attributes using biomedical ontologies [23-26]. Kumar et al. [23] used association rules to indicate dependence relationships between Gene Ontology terms using an annotation dataset and background knowledge. Myhre et al. [24], on the other hand, have focused entirely on proposing an additional gene ontology layer via discovering cross-ontology association rules from GO annotations. However, none of these approaches use the biomedical ontologies and, in particular, their hierarchical structure to compute interestingness measures. Another set of existing research on applying association rule mining to biomedical ontologies includes studies on mining single level, multi-level and cross-ontology association rules [27-29]. Carmona-Saez et al. [27], for example, mine single level associations between GO annotations and expressed genes from microarray data integrated with GO annotation information. However, as in the previous case, the inherent information provided by the ontology structure is not considered when computing the interestingness measures, and hence limit, to some extent, the knowledge discovered.

Interestingness measures play an essential role by reducing the number of discovered rules and retaining only those with the best utility, in a post-processing step. Different rule interestingness measures have different qualities or flaws. There is no optimal measure and one way to solve this challenge is to try to find a good compromise. Research has been performed on finding optimal measures for different datasets [8,9], but by taking into account only traditional interestingness measures.

In summary, prior efforts in association rule mining applied to datasets annotated with biomedical ontology concepts focus on mining normal, cross-ontology and multi-level association rules, but leave out the use of the semantic relationships between the target concepts from the computation of the interestingness measures.

### Conclusion

Concepts defined and described by biomedical ontologies, e.g., the Human Phenotype Ontology, enable us to compare medical terms at a semantic level – a comparison that is otherwise not possible. Our research has focused on the use of semantic relationships between patient phenotypes, annotated by HPO entities, in the process of mining association rules. In this manuscript, we have proposed a method that integrates concept similarity metrics into the computation of traditional interestingness measures, with application to finding association rules in the bone dysplasia domain. This method has been applied on an annotated patient dataset and used domain-specific semantic similarities.

Experimental results have led to the conclusion that, for our domain, Confidence is the most accurate measure, independently on the underlying computation method, i.e., traditional or semantic. On the other hand, Semantic Confidence was able to take advantage of structure of the domain ontologies and of the custom semantic similarity to achieve better results (up to 6.85% better accuracy over the traditional Confidence). In conclusion, these results suggest that, given an appropriate domain-specific ontology, semantic similarities are able to improve the efficiency of traditional interestingness measures in the association rule discovery process, hence enabling a valuable semantic interestingness measures framework.

## Competing interests

The authors declare that they have no competing interests.

## Authors’ contributions

JH and AZ formulated the basic idea behind SKELETOME. JH coordinates the project. TG leads the development of the project. RP and TG designed the experiments. RP run the experiments. RP and TG analysed the experimental results. AZ provided the domain expertise. RP and TG wrote the manuscript. JH and AZ edited the manuscript. All authors read and approved the final manuscript.
